# The effect of air pulse-driven whole eye motion on the association between corneal hysteresis and glaucomatous visual field progression

**DOI:** 10.1038/s41598-018-21424-8

**Published:** 2018-02-14

**Authors:** Shuichiro Aoki, Hiroshi Murata, Masato Matsuura, Yuri Fujino, Shunsuke Nakakura, Yoshitaka Nakao, Yoshiaki Kiuchi, Ryo Asaoka

**Affiliations:** 1 Department of Ophthalmology, The University of Tokyo, Tokyo, Japan; 2 Department of Rehabilitation, Orthopic and Visual Science, School of Allied Health Sciences, Kitasato University, Tokyo, Kanagawa Japan; 3 Department of Ophthalmology, Saneikai Tsukazaki Hospital, Himeji, Japan; 4 Department of Ophthalmology and Visual Sciences, Hiroshima University, Higashihiroshima, Japan

## Abstract

Corneal hysteresis (CH) measured with Ocular Response Analyzer (Reichert: ORA) has been reported to be closely related to the glaucomatous visual field (VF) progression. The air pulse applied to an eye not only induces corneal deformation, but also whole eye motion (WEM), which may result in an inaccurate measurement of CH. Here we investigated the influence of air pulse-driven WEM measured with the Corivs ST (CST^®^, OCULUS) on the relationship between CH and VF progression in primary open angle-glaucoma patients. Using the CST parameters of the maximal WEM displacement (WEM-d) and the time to reach that displacement (WEM-t), the eyes were classified into subgroups (WEM-d low- and high-group, and WEM-t short- and long-group). For the whole population and all subgroups, the optimal linear mixed model to describe mean of total deviation (mTD) progression rate with eight reliable VFs was selected from all combinations of seven parameters including CH. As a result, optimal models for the mTD progression rate included CH in the whole population, the WEM-d low- group and the WEM-t short-group, but not in the WEM-d high-group and the WEM-t long-group. Our findings indicated association between CH and glaucomatous progression can be weakened because of large WEM.

## Introduction

Corneal hysteresis (CH) measured with Ocular Response Analyzer (Reichert: ORA) is closely related to the progression of glaucomatous visual field (VF) damage^1–4^. It reflects corneal damping capacity in response to air pressure-induced stress^5,6^. In the ORA measurement, an air pulse induces external stress to deflect the cornea and CH is the difference of pressures at two applanation events during inward and outward movements. However, the air pulse can drive not only corneal deflection, but also whole eye motion, which could result in an inaccurate CH measurement; the ORA-CH measurement validity relies on an assumption that the majority of energy generated by the air pulse contributes to corneal deflection but if this is not the case due to whole eye motion, it could result in an inaccurate measurement of CH and also a weakened relationship between the measured CH value and the progression of glaucomatous VF damage.

The Corvis ST (OCULUS: CST, Ver 1.13b1361) provides various morphological and dynamic parameters of corneal deformation in response to an air-pulse pressure^7,8^. Recorded image sequences reveal that the air puff induces corneal backward deflection and, simultaneously, posterior movement of the whole cornea called “Whole Eye Motion (WEM)”; the WEM is summarized by two measurements of the maximal displacement (WEM-d) and the time taken to reach the maximal displacement (WEM-t). Thus, hypothesizing the relationship between the ORA-measured CH value and the progression of glaucomatous VF damage relies on the WEM, because the accuracy of the measurement of CH value depends on WEM, the main purpose of this study was to investigate the influence of these WEM parameters on the relationship between CH and glaucomatous VF progression. Further, since mechanical conditions in orbit is known to affect WEM^9^, we also investigated the effect of topical usage of PGA prescribed for glaucoma patients on WEM along with other parameters because PGA induces histological and mechanical changes in not only orbital tissue but also eyelids^10–12^, cornea and sclera^13–15^.

## Method

The study was approved by the Research Ethics Committee of the Graduated School of Medicine and Faculty of Medicine at The University of Tokyo. Written informed consent was given by patients for their information to be stored in the hospital database and used for research. The study was performed according to the tenets of the Declaration of Helsinki.

### Patients

108 eyes of 70 primary open angle-glaucoma patients (37 males and 33 females) were included. All patients had at least eight reliable VFs measured with the Humphrey Field Analyzer II (HFA, Carl Zeiss Meditec Inc, Dublin, CA), with the 24-2 or 30-2 SITA standard program. Reliable VFs were defined as Fixation loss (FL) rate <20% and False positive (FP) rate <15% following the criteria used in the HFA software; false negative (FN) rate was not used as an exclusion criterion. All patients had undergone a VF measurement prior to observation in the current study. We chose a minimum of eight VFs because it has recently been reported that this number is needed to precisely analyze VF progression^16–20^. Eyes that experienced any surgical procedure, including trabeculectomy and cataract surgery, during or prior to this VF series period were excluded. Inclusion criteria were no abnormal eye-related findings except for OAG on biomicroscopy, gonioscopy and funduscopy. Eyes with a history of other ocular disease, such as age-related macular degeneration were also excluded. Only subjects aged ≧20 years old were included and contact lens wearers were excluded. The mean and standard deviation (SD) of all Goldmann applanation tonometry based-intraocular pressure (GAT-IOP) measurements during the follow up period were calculated. Axial length (AL) and central corneal thickness (CCT) were also measured in all patients using the IOL Master, ver. 5.02 (Carl Zeiss Meditec, CA) and CST, respectively.

### VF data

The mean total deviation (mTD) value of the 52 test points in the 24-2 HFA VF test pattern was calculated. The progression rate of mTD was determined with linear regression analysis using the eight VFs collected from each eye, similarly to the MD trend analysis employed in the HFA Guided Progression Analysis (GPA).

### ORA measurements

ORA records two applanation pressure measurements, prior to and following an indentation of the cornea with the application of a rapid air jet. Due to its viscoelastic property, the cornea resists the air puff, resulting in delays in the inward and outward applanation events, which causes a measurable difference in the air puff values. This difference is called CH. ORA measurements were carried out three times with at least a five minutes’ interval between each measurement. ORA measurements were taken prior to, but on the same day of the GAT-IOP measurement, and also within 180 days from the eighth VF measurement. The order of ORA and CST measurements was decided randomly. All data had a quality index >7.5. In the current study, the average values of the three measured values of CH and corneal compensated IOP (IOPcc), but not corneal resistant factor (CRF), were used for analysis since previous studies have indicated CH, but not CRF, is associated with glaucomatous progression^1–4^.

### Corvis ST tonometry measurement

**Figure 1:**
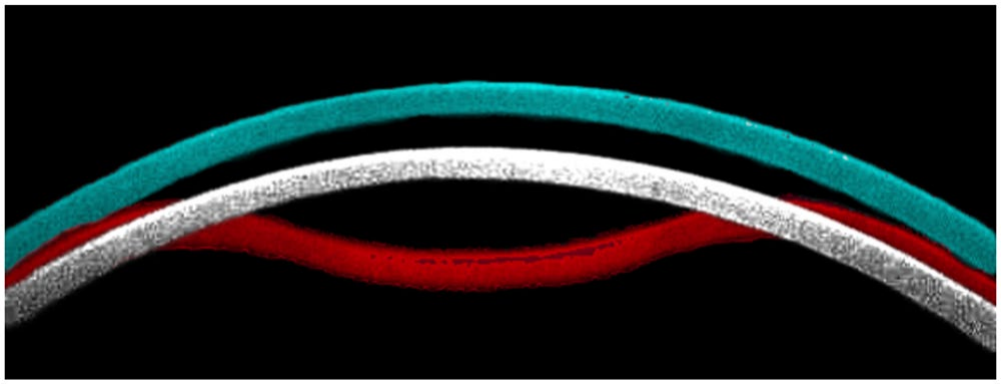
An illustration of corneal apical movement and ‘Whole Eye Motion’ in CST measurement. Corneal locations at the maximum backward displacement, or Whole Eye Motion (white, at 22.41 ms from the initiation of air jet) and maximum deformation concavity (red, at 16.40 ms from the initiation of air jet)) are superimposed on the cornea prior to the CST measurement (blue). In this case, the time of the second applanation (‘A2 deformation time’) was 22.05 ms. AICc: corrected Akaike Information Criterion, SE: standard error, CST: CorvisST, WEM-d: maximum whole eye motion displacement, WEM-t: a time needed for maximum whole eye motion displacement, mTD: mean of total deviations, CCT: central corneal thickness, CH: corneal hysteresis, IOPcc: corneal compensated IOP, AL: axial length, GAT-IOP: Goldmann applanation tonometry-based intraocular pressure.

The principles of CST are described in detail elsewhere^8^. The instrument’s ultra-high speed camera, capturing 4,330 images per second, records 140 images of corneal deformation during the 30 ms air puff. The device provides the same load over the same time period, ensuring reliable quantification and comparison between eyes. The air pressure induces not only corneal deflection from which various corneal deformation-related parameters like deformation amplitude, applanation length or corneal velocity are provided, but also a backward movement of the whole cornea, the amount of which is parametrized as the WEM. Maximum WEM which usually takes place near the event of the second applanation is identified from the image sequence and the maximal displacement (WEM-d [mm]) and the time (WEM-t [ms]) to reach the maximal dislocation are calculated (see Fig. 1).

CST (software version; ver. 1.3r1512) was performed three times on the same day with ORA measurement, with at least a five minutes’ interval between each measurement. Only reliable CST measurements, according to the “OK” quality index displayed on the device monitor, were used. The average values of the three measured values of WEM-d, WEM-t and CST-measured IOP (CST-IOP) were calculated and used in the analysis. CCT was also measured with the CST and used in the analyses.

### Prostaglandin analogues usage assessment

Every patient’s medical history was surveyed from the electronical medical record and the usage of a prostaglandin analogue (PGA) eyedrop was identified and eyes were categorized into either of “PGA usage group” or “PGA non-usage group”.

### Statistical analysis

First, the relationships between WEM-d or WEM-t and CH and other ocular/systemic parameters (age, gender, CCT, CH, AL, initial mTD, GAT-IOP on the same date, and mTD progression rate) were calculated using the linear mixed model. Then, from all eyes, the average values of WEM-d and WEM-t were calculated and eyes were divided into two subpopulations in two ways; WEM-d low-group: eyes with WEM-d lower than the average value, WEM-d high-group: eyes with WEM-d higher than the average value, and also WEM-t short-group: eyes with WEM-t shorter than the average value, WEM-t long-group: eyes with WEM-t longer than the average value. Then, for each subgroup, the association between mTD progression rate and the seven variables of CH and other ocular/systemic parameters (age, mean GAT-IOP, SD of GAT-IOP, CCT, AL, and mTD in the initial VF) was investigated using a linear mixed model, where the patient was registered as a random effect (because one or two eyes of a patient were included in the current study). The optimal linear mixed model to describe mTD progression rate was selected according to the second order bias corrected Akaike Information Criterion (AICc) index from all possible combinations of predictors (2^7^ patterns). The AICc is the corrected form of the common statistical measure of AIC that gives an accurate estimation even when the sample size is small^21^. Any magnitude of reduction in AICc suggests an improvement of the model^22,23^. The relative likelihood that one particular model is minimizing information loss compared to the model with the smallest AICc is calculated as exp((AICmin − AICx)/2), where AICx is AICc value of arbitrary model “X” and AICmin is the minimum value from all possible models^24^. In addition, further model selection seeking for an optimal model for mTD progression rate was conducted for all eyes and each subgroup, including IOPcc and CST-IOP, in addition to the above described seven parameters. In other words, the model selection was carried out amongst 2^9^ patterns including IOPcc or CST-IOP.

All statistical analyses were performed using the statistical programming language ‘R’ (R version 3.2.3; The foundation for Statistical Computing, Vienna, Austria).

### Data Availability

The datasets analysed during the current study are available from the corresponding author on reasonable request.

## Results

**Figure 2:**
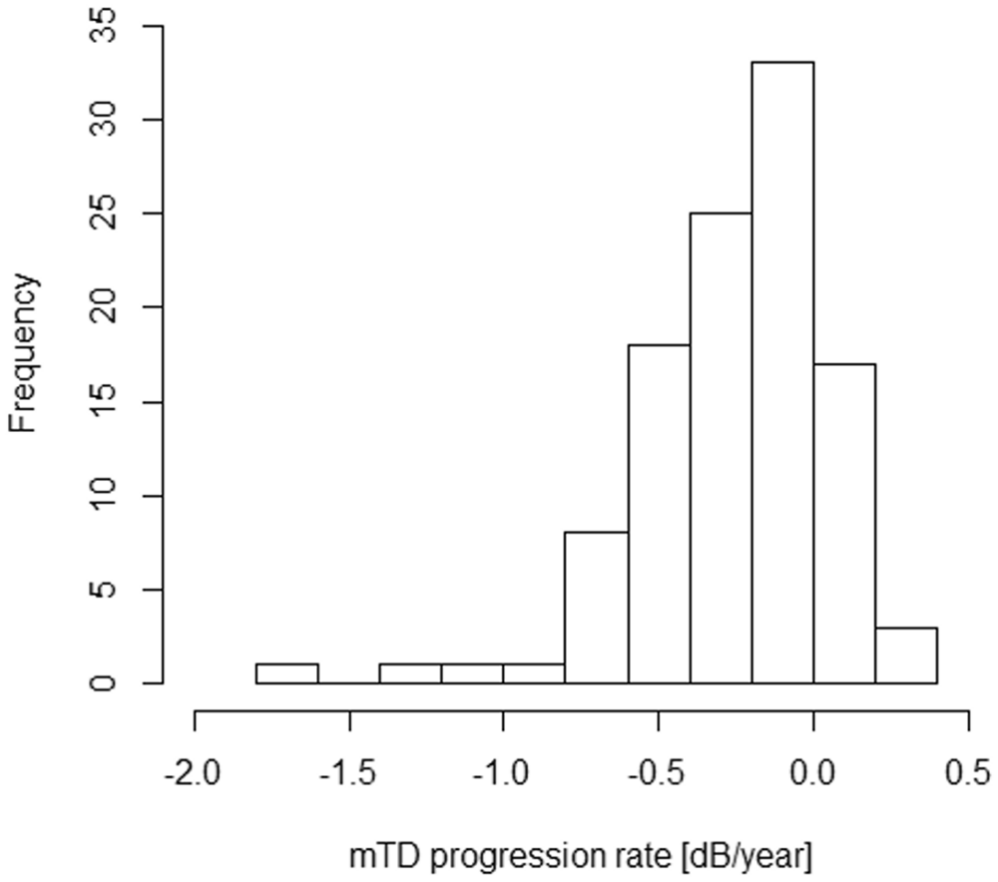
Histogram of mTD progression rate. mTD progression rate was −0.25 ± 0.32 [−1.78 to 0.27] dB/year. AICc: corrected Akaike Information Criterion, SE: standard error, CST: CorvisST, WEM-d: maximum whole eye motion displacement, WEM-t: a time needed for maximum whole eye motion displacement, mTD: mean of total deviations, CCT: central corneal thickness, CH: corneal hysteresis, IOPcc: corneal compensated IOP, AL: axial length, GAT-IOP: Goldmann applanation tonometry-based intraocular pressure.

Characteristics of the patients as well as the CH, WEM-d and WEM-t values are summarized in Table 1. The mean ± standard deviation (SD) [range] of age was 53.91 ± 10.23 [32.68 to 79.00]. Eight VFs were measured over the period of 2257.3 ± 983.6 [371 to 6895] days and GAT-IOP was conducted 29.0 ± 7.1 [18 to 69] times during this period. Mean GAT-IOP was 13.5 ± 2.2 [8.9 to 20.2] mmHg. As shown in Fig. 2, mTD progression rate was −0.25 ± 0.32 [−1.78 to 0.27] dB/year. 55 eyes were in the WEM-d low-group and 53 eyes were in the WEM-d high-group, whereas 54 eyes were in the WEM-t short-group and 54 eyes were in the WEM-t long-group. Baseline characteristics of these subgroups are shown in Table 2 and Table 3. There was no significant difference in six variables (age, mean GAT-IOP, CCT, initial mTD, CH) between WEM-d low-group and high-group (p = 0.31 to 0.76, linear mixed model), and between WEM-t short- and long-group (p = 0.63 to 1.00, linear mixed model). There was a significant relationship between WEM-d and WEM-t (p = 0.021, linear mixed model; see Table 4).

**Table 1:** Summary of basic demographics.

Variables	Value
age, (mean ± SD) [range], years old	53.91 ± 10.23 [32.68 to 79.00]
Male/Female	37/33
Right/Left	56/52
GAT-IOP, (mean ± SD) [range], mmHg	13.47 ± 2.26 [8.93 to 20.19]
AL, (mean ± SD) [range], mm	25.13 ± 1.64 [22.30 to 29.20]
CCT, (mean ± SD) [range], μm	530.9 ± 35.82 [458.3 to 624.3]
Initial mTD, (mean ± SD) [range], dB	−5.72 ± 5.44 [−22.44 to 2.48]
WEM-d low group/WEM-d high group	55/53
WEM-t short group/WEM-t long group	54/54
CH, (mean ± SD) [range], mmHg	9.16 ± 1.14 [6.50 to 11.79]
WEM-d, (mean ± SD) [range], mm	0.31 ± 0.071 [0.16 to 0.46]
WEM-t, (mean ± SD) [range], ms	22.42 ± 0.78 [19.98 to 23.90]

AICc: corrected Akaike Information Criterion, SE: standard error, CST: CorvisST, WEM-d: maximum whole eye motion displacement, WEM-t: a time needed for maximum whole eye motion displacement, mTD: mean of total deviations, CCT: central corneal thickness, CH: corneal hysteresis, IOPcc: corneal compensated IOP, AL: axial length, GAT-IOP: Goldmann applanation tonometry-based intraocular pressure.

**Table 2:** Summary of basic demographics of WEM-d low- and high-group.

Variables	WEM-d low-group	WEM-d high-group
age, (mean ± SD) [range], years old	51.18 ± 8.33 [32.68 to 67.00]	56.75 ± 11.28 [34.32 to 79.00]
GAT-IOP, (mean ± SD) [range], mmHg	13.39 ± 2.34 [9.91 to 20.19]	13.56 ± 2.19 [8.93 to 19.23]
AL, (mean ± SD) [range], mm	25.63 ± 1.42 [22.74 to 29.2]	24.60 ± 1.70 [22.30 to 28.76]
CCT, (mean ± SD) [range], μm	527.0 ± 35.87 [458.7 to 624.0]	534.6 ± 36.78 [480.7 to 624.7]
Initial mTD, (mean ± SD) [range], dB	−5.26 ± 5.21 [−22.44 to 2.00]	−6.20 ± 5.68 [−20.00 to 2.48]
CH, (mean ± SD) [range], mmHg	9.00 ± 1.09 [7.08 to 11.79]	9.32 ± 1.17 [6.50 to 11.72]

AICc: corrected Akaike Information Criterion, SE: standard error, CST: CorvisST, WEM-d: maximum whole eye motion displacement, WEM-t: a time needed for maximum whole eye motion displacement, mTD: mean of total deviations, CCT: central corneal thickness, CH: corneal hysteresis, IOPcc: corneal compensated IOP, AL: axial length, GAT-IOP: Goldmann applanation tonometry-based intraocular pressure.

**Table 3:** Summary of basic demographics of WEM-t short- and long-group.

Variables	WEM-t short-group	WEM-t long-group
age, (mean ± SD) [range], years old	54.44 ± 10.75 [32.68 to 74.42]	53.38 ± 9.75 [34.32 to 79.00]
GAT-IOP, (mean ± SD) [range], mmHg	14.25 ± 2.03 [10.14 to 10.23]	12.69 ± 2.23 [8.93 to 20.19]
AL, (mean ± SD) [range], mm	25.07 ± 1.70 [22.30 to 28.76]	25.19 ± 1.59 [22.74 to 29.20]
CCT, (mean ± SD) [range], μm	538.4 ± 30.51 [486.0 to 624.7]	523.1 ± 40.23 [458.7 to 624.0]
Initial mTD, (mean ± SD) [range], dB	−5.54 ± 5.54 [−22.44 to 2.48]	−5.90 ± 5.38 [−20.00 to 2.00]
CH, (mean ± SD) [range], mmHg	9.16 ± 1.10 [6.50 to 11.72]	9.16 ± 1.18 [7.08 to 11.79]

AICc: corrected Akaike Information Criterion, SE: standard error, CST: CorvisST, WEM-d: maximum whole eye motion displacement, WEM-t: a time needed for maximum whole eye motion displacement, mTD: mean of total deviations, CCT: central corneal thickness, CH: corneal hysteresis, IOPcc: corneal compensated IOP, AL: axial length, GAT-IOP: Goldmann applanation tonometry-based intraocular pressure.

**Table 4:** The relationship between the values of WEM-d or WEM-t and age, gender, CCT, CH, AL, and GAT-IOP on the measurement day, with the p values based on linear mixed model.

	WEM-d (mm)			WEM-t (ms)		
	Coefficient	SE	p value	Coefficient	SE	p value
Age	0.0017	0.00076	0.027	−0.0020	0.0086	0.82
Gender	−0.020	0.017	0.23	−0.14	0.18	0.45
CCT	0.000039	0.00023	0.87	−0.0031	0.0025	0.22
CH	0.0065	0.0062	0.30	0.0077	0.069	0.91
AL	−0.016	0.0044	0.00061	0.016	0.053	0.76
GAT-IOP	0.0010	0.0027	0.71	−0.037	0.030	0.22
WEM-d	—	—	—	2.53	1.05	0.021

AICc: corrected Akaike Information Criterion, SE: standard error, CST: CorvisST, WEM-d: maximum whole eye motion displacement, WEM-t: a time needed for maximum whole eye motion displacement, mTD: mean of total deviations, CCT: central corneal thickness, CH: corneal hysteresis, IOPcc: corneal compensated IOP, AL: axial length, GAT-IOP: Goldmann applanation tonometry-based intraocular pressure.

**Figure 3:**
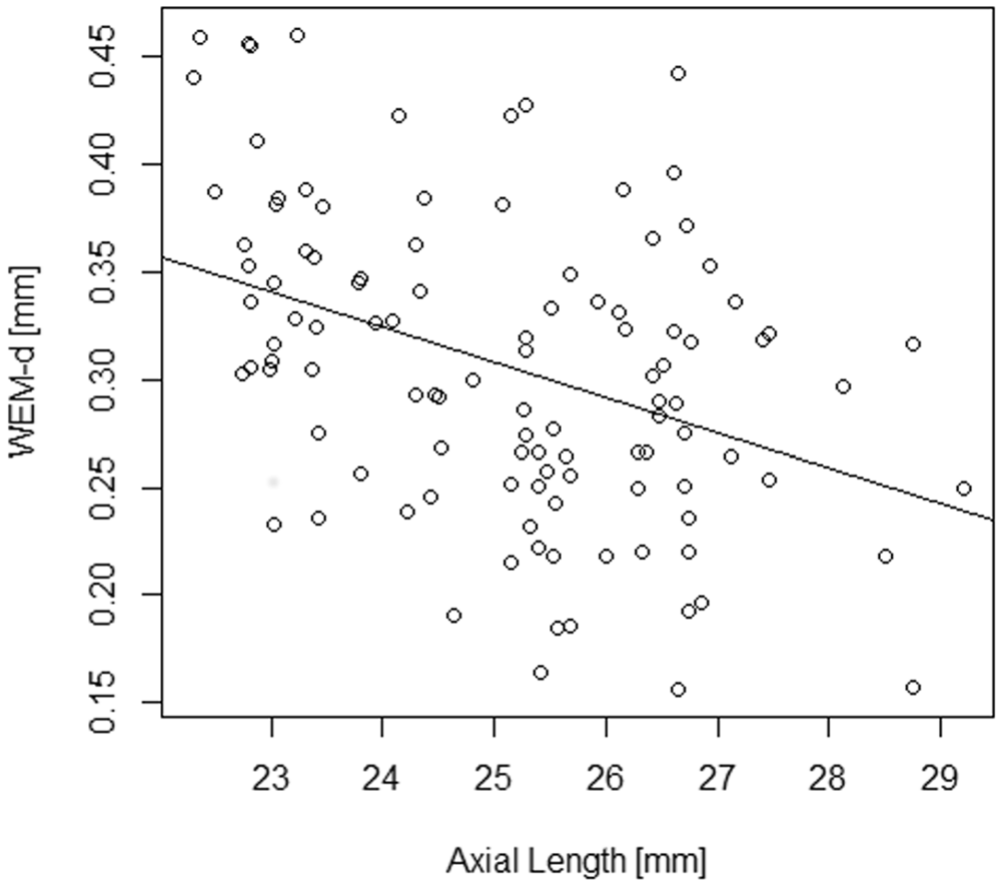
The relationship between WEM-d and AL. WEM-d was significantly related to AL in all eyes. AICc: corrected Akaike Information Criterion, SE: standard error, CST: CorvisST, WEM-d: maximum whole eye motion displacement, WEM-t: a time needed for maximum whole eye motion displacement, mTD: mean of total deviations, CCT: central corneal thickness, CH: corneal hysteresis, IOPcc: corneal compensated IOP, AL: axial length, GAT-IOP: Goldmann applanation tonometry-based intraocular pressure.

The relationships between WEM-d or WEM-t and age, gender, CH, CCT, AL, GAT-IOP on the same measurement day, and mTD progression rate are shown in Table 4. WEM-d was significantly related to age and AL (p = 0.027 and 0.00061, linear mixed model); see Fig. 3. WEM-d and WEM-t had no significant relationship with the other variables, including CH (p = 0.30 and 0.91, respectively, linear mixed model).

**Figure 4:**
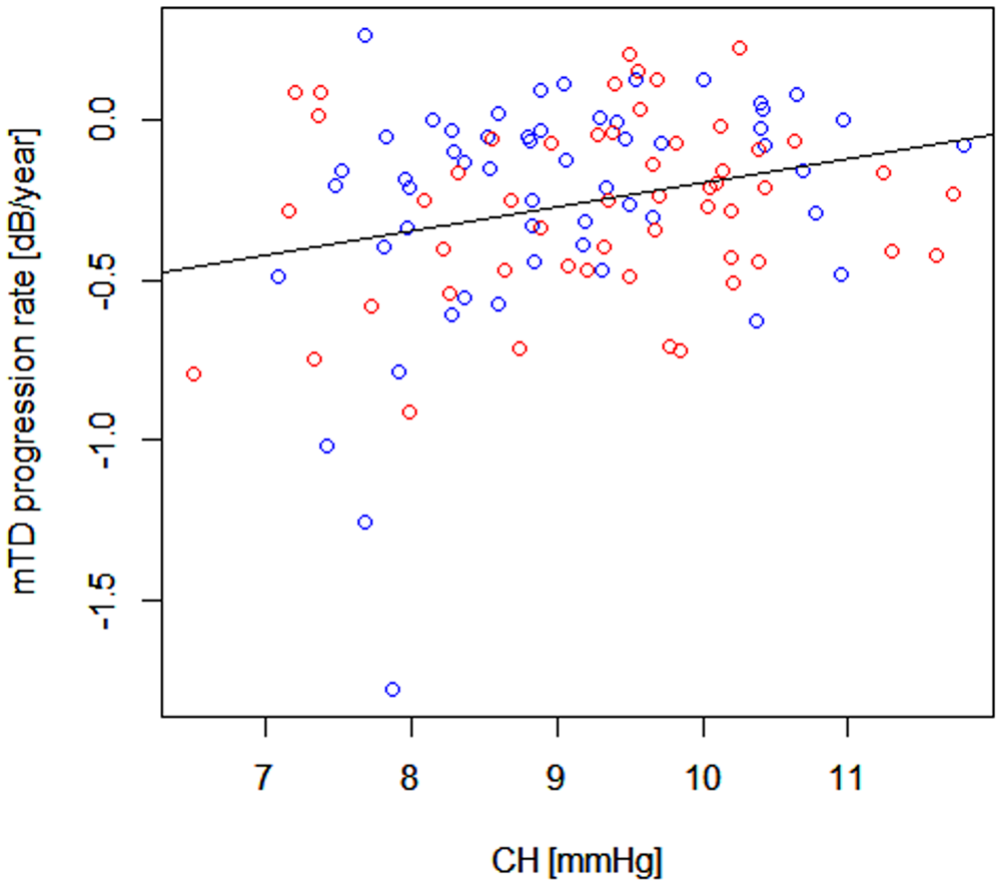
The relationship between mTD progression rate and CH. mTD progression rate was significantly related to CH in all eyes. AICc: corrected Akaike Information Criterion, SE: standard error, CST: CorvisST, WEM-d: maximum whole eye motion displacement, WEM-t: a time needed for maximum whole eye motion displacement, mTD: mean of total deviations, CCT: central corneal thickness, CH: corneal hysteresis, IOPcc: corneal compensated IOP, AL: axial length, GAT-IOP: Goldmann applanation tonometry-based intraocular pressure.

The relationships between mTD progression rate and age, gender, CH, CCT, AL, WEM-d, WEM-t and mean GAT-IOP are shown in Table 5. Mean GAT-IOP was not significantly related to mTD progression rate in all eyes, or any of the subgroups (WEM-d low, WEM-d high, WEM-t short and WEM-t long groups; p = 0.22, 0.77, 0.12, 0.12 and 0.86, respectively, linear mixed model). As shown in Fig. 4, CH was significantly related to mTD progression rate in all eyes (mTD progression rate = −0.94 + 0.075 × CH. p = 0.011, linear mixed model). This significant relationship was also observed in the WEM-d low group (mTD progression rate = −1.20 + 0.11 × CH. p = 0.034, linear mixed model), however, it was not observed in the WEM-d high group (p = 0.22), WEM-t short group (p = 0.088) or the WEM-t long group (p = 0.085).

**Table 5:** The relationship between the values of mTD progression rate and age, gender, CCT, CH, AL, WEM-d, WEM-t, and mean GAT-IOP, with the p values based on linear mixed model.

	mTD progression rate		
	Coefficient	SE	p value
Age	−0.0043	0.0033	0.19
Gender	0.0073	0.067	0.91
CCT	0.0015	0.00092	0.11
CH	0.075	0.028	0.011
AL	−0.0026	0.020	0.90
WEM-d	0.36	0.46	0.44
WEM-t	0.030	0.042	0.47
Mean GAT-IOP	0.019	0.015	0.22

AICc: corrected Akaike Information Criterion, SE: standard error, CST: CorvisST, WEM-d: maximum whole eye motion displacement, WEM-t: a time needed for maximum whole eye motion displacement, mTD: mean of total deviations, CCT: central corneal thickness, CH: corneal hysteresis, IOPcc: corneal compensated IOP, AL: axial length, GAT-IOP: Goldmann applanation tonometry-based intraocular pressure.

As shown in Table 6, in all eyes (108 eyes), the optimal model describing mTD progression rate was: mTD progression rate = −0.95 + 0.075 × CH (AICc = 55.79); adding other variables did not improve the model as suggested by the decrease of AICc. In the WEM-d low-group, the optimal model describing mTD progression rate was: mTD progression rate = −1.20 + 0.11 × CH (AICc = 42.78). In the WEM-d high-group, the optimal model describing mTD progression rate was: mTD progression rate = −0.64 + 0.028 × mean GAT-IOP (AICc = 17.66). In this group, the model included only CH as an independent variable; the AICc value was equal to 18.70 (mTD progression rate = −0.65 + 0.041 × CH). In the WEM-d high-group, the relative likelihood that the smallest AICc model was the optimal model compared to the CH monovariate model was calculated as 1.68.

**Table 6:** The optimal models and CH monovariate models in each subgroup.

Group	Variables in optimal model (coefficient ± SE, p value)	AICc of optimal model	AICc of CH monovariate model
All eye (108 eyes)	CH (0.075 ± 0.028, p = 0.011)	55.79	55.79
WEM-d low-group (55 eyes)	CH (0.11 ± 0.045, p = 0.034)	42.78	42.78
WEM-d high-group (53 eyes)	mean GAT-IOP (0.028 ± 0.017, p = 0.12)	17.66	18.70
WEM-t short-group (54 eyes)	mean GAT-IOP (0.038 ± 0.024, p = 0.14)	40.18	40.38
	CH (0.080 ± 0.045, p = 0.10)		
WEM-t long-group (54 eyes)	Age (−0.012 ± 0.0038, p = 0.047)	17.39	23.90
	Initial mTD (0.012 ± 0.0068, p = 0.10)		

SE: standard error, AICc: corrected Akaike Information Criterion, SE: standard error, CST: CorvisST, WEM-d: maximum whole eye motion displacement, WEM-t: a time needed for maximum whole eye motion displacement, mTD: mean of total deviations, CCT: central corneal thickness, CH: corneal hysteresis, IOPcc: corneal compensated IOP, AL: axial length, GAT-IOP: Goldmann applanation tonometry-based intraocular pressure.

In the WEM-t short-group, the optimal model describing mTD progression rate was: mTD progression rate = −1.54 + 0.038 × mean GAT-IOP + 0.080 × CH (AICc = 40.18). In this group, the model including only CH as an independent variable had an AICc value of 40.38 (mTD progression rate = −1.05 + 0.085 × CH), which had the second smallest AICc across all possible 2^7^ models. In the WEM-t long-group, the optimal model describing mTD progression rate was: mTD progression rate = 0.51 − 0.012 × age + 0.012 × Initial mTD (AICc = 17.39). In this group, the model including only CH as an independent variable had an AICc value of 23.90, which was, again, the second smallest across all possible models. The relative likelihood that the smallest AICc model was the optimal model compared to the CH monovariate model calculated was 1.10 and 25.9 for WEM-t short-group and WEM-t long group, respectively.

Further model selection seeking for an optimal model for mTD progression rate, including IOPcc and CST-IOP as covariates, resulted in a different optimal model only in WEM-t low group. This optimal model was: mTD progression rate = −0.59 + 0.075 × mean GAT-IOP −0.048 × IOPcc (AICc = 39.23). The same variables were selected in all eyes and the other subgroups as shown in Table 6.

As shown in Table 7, 65 eyes were in the PGA usage group and 43 eyes were in the PGA non-usage group. Initial mTD was significantly different between these groups (p = 0.03, linear mixed model). The WEM-d value in the PGA usage and PGA non-usage groups were 0.300 ± 0.060 [0.164 to 0.443] and 0.314 ± 0.085 [0.156 to 0.461], respectively; these were not significantly different (linear mixed model, p = 0.12). The WEM-t value in the PGA usage and PGA non-usage groups were 22.48 ± 0.78 [20.14 to 23.90] and 22.34 ± 0.77 [19.98 to 23.87], respectively; these were again not significantly different (linear mixed model, p = 0.24). These differences remained non-significant after including age and AL as a covariate (p = 0.83 and 0.23, respectively). There was no significant difference in CH (p = 0.34) or the other variables between the two groups (Table 7).

**Table 7:** Comparisons of parameters between PGA usage and non-usage groups.

	PGA usage group (65 eyes)	PGA non-usage group (43 eyes)	P value
age, years old	51.48 ± 9.6 [32.7 to 74.4]	57.58 ± 10.1 [37.4 to 79.0]	0.29
Mean GAT-IOP, mmHg	13.3 ± 2.1 [8.9 to 18.3]	13.7 ± 2.49 [9.4 to 20.2]	0.40
SD of GAT, mmHg	1.6 ± 0.43 [0.83 to 2.8]	1.5 ± 0.52 [0.79 to 3.6]	0.41
AL, mm	25.3 ± 1.4 [22.7 to 28.1]	24.9 ± 1.9 [22.3 to 29.2]	0.35
CCT, µm	532.6 ± 38.6 [458.3 to 624.3]	528 ± 31.5 [462.0 to 622.3]	0.23
Initial mTD, dB	−6.5 ± 5.5 [−20.0 to 0.52]	−4.5 ± 5.2 [−22.4 to 2.5]	0.030
mTD progression rate, dB/year	−0.23 ± 0.29 [−1.26 to 0.23]	−0.28 ± 0.36 [−1.8 to 0.27]	0.42
CH, mmHg	9.2 ± 1.1 [7.08 to 11.7]	9.1 ± 1.2 [6.5 to 11.8]	0.34
WEM-d, mm	0.30 ± 0.060 [0.16 to 0.44]	0.31 ± 0.085 [0.16 to 0.46]	0.66
WEM-t, ms	22.5 ± 0.78 [20.1 to 23.9]	22.3 ± 0.77 [20.0 to 23.9]	0.24

Values are shown as mean ± SE [range]. P values were obtained using the linear mixed model. AICc: corrected Akaike Information Criterion, SE: standard error, CST: CorvisST, WEM-d: maximum whole eye motion displacement, WEM-t: a time needed for maximum whole eye motion displacement, mTD: mean of total deviations, CCT: central corneal thickness, CH: corneal hysteresis, IOPcc: corneal compensated IOP, AL: axial length, GAT-IOP: Goldmann applanation tonometry-based intraocular pressure.

## Discussion

In the current study, ORA and CST measurements were conducted in 108 eyes of 70 patients with POAG. As a result, CH was significantly related to mTD progression rate in all eyes. We also investigated the relationship between CH and mTD progression rate, in the relationship with WEM. We observed that CH was significantly related to mTD progression rate in the WEM-d low group, however, this relationship was not significant in the WEM-d high, WEM-t short and WEM-t long-groups. Furthermore, in all eyes, as well as the WEM-d low and WEM-t short-groups, the optimal models describing mTD progression rate included CH, however, this was not the case for the WEM-d high and WEM-t long-groups. The usage of PG was not significantly related to WEM nor other parameters.

The associations between ORA-CH and both glaucomatous morphological and functional changes have been investigated in various previous studies. Some studies have reported that CH is associated with mean cup depth, cup-to-disc ratio, rim area, retinal nerve fiber layer average thickness and the acquired pit of the optic disc^25–27^. Others have reported CH is low in glaucomatous patients compared to non-glaucomatous eyes^28–32^. Furthermore, low CH has been reported to be associated with fast progression of glaucomatous VF and optic nerve changes^1–4^. As CH is the difference between pressures at two corneal applanation events, some studies argue that CH value reflects ‘damping capacity’ of cornea: i.e., function as stress absorber^9,33,34^. From this point of view, the ORA-CH measurement might be invalid if part of energy generated by the air pulse is converted to whole eye kinetic energy, resulting in an perturbed stress profile on cornea itself and inaccurate measurement of CH.

In the WEM-d high-group, as shown in Table 6, the optimal model included mean GAT-IOP, but not CH. The AICc value of the CH monovariate model in this group was higher than that of this optimal model by 1.04. In the WEM-d low-group, in contrast, the CH monovariate model itself had the minimum AICc value among all the possible models. On the other hand, in the WEM-t long-group, the optimal model included age and initial mTD. The AICc value of this optimal model was smaller by 6.51 than that of the CH monovariate model. In the WEM-t short-group, the AICc value of the CH monovariate model was higher than the optimal model, including mean GAT-IOP and CH, by 0.20. These findings suggest that larger WEM displacement (WEM-d) weakens the association between CH and glaucomatous VF progression because some proportion of the applied energy that does not only contribute to corneal deformation but also causes significant eye motion, which may counteract reliable CH measurement. Also, a larger time taken to reach the maximal displacement (WEM-t) may cause the consumption of more energy as the result of friction between the eye and orbit.

Inclusion of IOPcc and CST-IOP in the model selection did not alter selected variables in the optimal model in all eyes, WEM-d low-group, WEM-d high-group, and WEM-t long-group. On the other hand, in WEM-t short-group, IOPcc and mean GAT-IOP were included (AICc = 39.23) instead of CH (AICc = 40.18). This is probably because IOPcc is closely associated with CH^35,36^, but also associated with IOP level at the time of measurement (Asaoka R, *et al*. IOVS. 2008;49: ARVO E-Abstract E703). Thus, these results suggest that mTD progression rate was best described by a well-established risk factor of GAT-IOP^37–41^, in conjunction with CH and IOP at the time of ORA measurement, in WEM-t short-group. On the contrary, in WEM-d low-group, the optimal model for mTD progression rate remained including CH. This may be because WEM-d and -t are related to IOP level at the time of measurement, so we analyzed the relationship between these WEM related parameters and three IOPs (IOPcc, CST-IOP and GAT-IOP) on the day of CST measurement. However, none of these were significantly related (p > 0.05, linear mixed model, data not shown in Result).

Previous large population studies established GAT-IOP^37–41^, age^38–41^, and VF damage at treatment initiation^38^ as predictive factors for glaucomatous VF progression. Among these established risk factors, in the current study, mean GAT-IOP was included in the optimal model for mTD progression rate in the WEM-d high-group, and age and initial mTD were included in the optimal model for mTD progression rate in the WEM-t long group, whereas CH was not included in the optimal models in these groups (see Table 6). This is probably because, CH was not accurately measured because of large WEM, and instead these other variables were selected.

There was not a significant relationship between CH and WEM-d or WEM-t in the current study. CH was evaluated with ORA whereas WEM was measured with CST. In the ORA measurement, the magnitude of the air jet applied to the cornea is proportional to IOP in ORA (P1 pressure). On the other hand, air pressure is of a uniform magnitude (70 mmHg) for all eyes, irrespective of the IOP level, in the CST measurement. Thus, a tighter relationship may be observed between CH and WEM, if the latter was measured using ORA. Also, despite its name, the WEM in CST is a measurement of the cornea, which is not necessarily identical to genuine motion of whole eye, because the shapes of eye balls are different from eye to eye. Thus, different results could be observed if the motion of the back of the eye ball was measured.

Topical usage of PGA induces various adverse effects around the eyelids, including deepening of the upper eyelid sulcus, flattening of the lower eyelid bags, orbital fat atrophy and a tight orbit, as collectively termed as ‘prostaglandin-associated periorbitopathy’^10–12^ As PGA induces a change in the connective tissue in orbit, it can also have some effects on the eye motion in the response to external stress. Further, PGA usage can alter the cornea and sclera. For instance, Harasymowycz *et al*. reported that the usage of travoprost decreased CCT thickness^42^. Moreover, the usage of PGA leads to upregulation of matrix metalloproteinases and downregulation of tissue inhibitors of matrix metalloproteinases associated with altered gene expression, as well as decreased collagen type I level and corneal thickness, in animal and human experiments^13–15^. The effects of these changes in the cornea, sclera and orbit on WEM are undoubtedly complex; the tight but fat atrophic orbit may influence WEM, but at the same time, a weakened and thin cornea would be more easily deformed by an applied external stress. In the current study, the WEM-d and WEM-t value were not significantly different between the PGA usage and non-usage groups. The current data was obtained from a real world clinic where PGA is usually prescribed in eyes with visual field progression. Thus, a future study is needed to further investigate the relationship between PGA usage and the movements of the whole eye, measuring whole eye movements before and after usage of PGA.

A limitation of the current study is that age was not matched between the PGA usage and non-usage groups. We statistically adjusted this effect in the current analyses, however, a further study would be needed to confirm the current results using age-matched groups.

In conclusion, the effect of eye motion on the relationship between CH and glaucomatous progression was investigated. As a result, the relationship between CH and glaucomatous visual field progression was weak in eyes with large WEM. PGA usage had no significant effects on WEM.
